# Archiving of Cardiac Catheterization Laboratory Procedures and Records of Radiation Exposure: A Single-Center Experience

**DOI:** 10.7759/cureus.119356

**Published:** 2026-11-09

**Authors:** Pranab Jyoti Bhattacharyya, Maitreyee Bhattacharyya, Jahir Alam Choudhury

**Affiliations:** 1 Cardiology, Cardiothoracic and Neurosciences Centre, Gauhati Medical College and Hospital, Guwahati, IND; 2 Cardiac Care Technology, Gauhati Medical College and Hospital, Guwahati, IND

**Keywords:** cumulative air kerma, dose area product, fluoroscopy time, procedural archiving, radiation exposure

## Abstract

Background and objective

Cardiac catheterization laboratories (Cath Labs) increasingly perform fluoroscopy-guided diagnostic and interventional procedures, making systematic monitoring of radiation exposure important. Structured archiving can support radiation surveillance, quality assurance, and procedural evaluation. This study aimed to examine dose-area product/kerma-area product (DAP/KAP), cumulative air kerma (CAK/Ka,r), and fluoroscopy time, with particular attention to variation by procedure type and the influence of BMI.

Methods

A hospital-based retrospective cohort study was conducted at the Cardiac Catheterization Laboratory of Gauhati Medical College and Hospital. A total of 190 archived patient records were analyzed. Demographic variables, procedure category, fluoroscopy time, DAP/KAP, and CAK were evaluated. The association between BMI and radiation exposure was also examined.

Results

The mean age of the cohort was 55.88 ± 11.04 years and mean BMI was 26.78 ± 3.87 kg/m². Coronary angiography (CAG) + percutaneous transluminal coronary angioplasty (PTCA) was the most frequently performed procedure (70.5%). Median DAP/KAP and CAK were lowest for diagnostic CAG and substantially higher for CAG + PTCA. The median fluoroscopy time was 1.9 minutes for CAG, 8.3 minutes for CAG + PTCA, and 13.1 minutes for other procedures. BMI demonstrated a significant positive correlation with DAP/KAP (r = 0.470, p < 0.001) and CAK (r = 0.469, p < 0.001). DAP/KAP and CAK demonstrated a very strong positive correlation (r = 0.941, p < 0.001). No radiation-related complications or dose alerts were documented.

Conclusions

Radiation exposure varied by procedure type and BMI. Systematic Cath Lab archiving and continuous monitoring of DAP/KAP, CAK, and fluoroscopy time can provide useful baseline data for radiation safety, quality assurance, and dose optimization.

## Introduction

Cardiovascular diseases remain a major cause of morbidity and mortality worldwide. The increasing use of invasive diagnostic and therapeutic procedures has made the cardiac catheterization laboratory (Cath Lab) an essential component of contemporary cardiovascular care [1-4]. Common procedures include coronary angiography (CAG), percutaneous transluminal coronary angioplasty (PTCA), electrophysiological procedures, and structural heart interventions. Fluoroscopic imaging provides real-time visualization of coronary anatomy, catheters, guidewires, balloons, and other therapeutic devices during these procedures [2,3,5].

However, fluoroscopy uses ionizing radiation and therefore exposes both patients and healthcare personnel to radiation. The radiation burden depends on procedure type and complexity, fluoroscopy duration, number of cine acquisitions, imaging settings, operator technique, and patient body habitus [5-7]. Radiation monitoring is therefore an important component of Cath Lab practice. Cardiac catheterization laboratories generate large volumes of procedural and radiation-related data. Structured archiving allows these data to be used for radiation surveillance, procedural evaluation, quality assurance, clinical auditing, performance benchmarking, and research [6,8-12]. Digital Cath Lab systems have further improved the accessibility and long-term analysis of procedural records.

We undertook a retrospective study at our centre to establish systematic documentation of Cath Lab procedures and radiation exposure parameters. The study examined dose-area product/kerma-area product (DAP/KAP), cumulative air kerma (CAK/Ka,r) and fluoroscopy time, with particular attention to variation according to procedure type and the influence of BMI. The primary objectives of our study were to archive various cardiac Cath Lab procedures and to record radiation exposure parameters, namely DAP/KAP, CAK/Ka,r, and fluoroscopy time. The secondary objectives were to evaluate the relationship between BMI and radiation exposure during fluoroscopy-guided cardiac procedures and to compare radiation burden among different procedures, namely CAG, CAG + PTCA, and other procedures (device closure and plain old balloon angioplasty (POBA)).

## Materials and methods

A hospital-based retrospective cohort study was conducted using archived Cath Lab records at the Department of Cardiology, Cardiothoracic and Neurosciences Centre, Gauhati Medical College and Hospital, Assam. The study period was six months, from December 1, 2025, to May 31, 2026. The study included consecutive case sampling, and 190 eligible patient records were included in the final analysis. Unfortunately, the initial screening log and reason-specific exclusion counts were not maintained during retrospective data collection; therefore, these figures could not be reliably reconstructed. Patients undergoing CAG, CAG + PTCA, POBA, and device closure were included. The final analytical dataset was reviewed for completeness, and no missing values were identified among the variables included in the analysis. All procedures were performed using Philips Allura Xper FD10 systems (Philips Healthcare, Amsterdam, Netherlands) according to departmental protocols. Variables included age, sex, height, weight, BMI, procedure category, fluoroscopy time, DAP/KAP, and CAK/Ka,r. Extracted information was cross-checked against the available original Cath Lab procedural records, radiation dose reports, and hospital medical records. Duplicate entries, missing variables, and inconsistent values were reviewed, and discrepancies were checked against source documentation wherever possible, and a study-specific digital archive was created using Microsoft Excel, comprising a standardized master chart of 190 patient records.

Data were entered and analyzed using SPSS Statistics for Windows, Version 26.0 (IBM Corp., Armonk, NY). Continuous variables were summarised using mean ± standard deviation (SD), median with interquartile range (IQR), and range (minimum-maximum), as appropriate. Categorical variables were expressed as frequencies and percentages. Associations between BMI and DAP/KAP, BMI and CAK, and DAP/KAP and CAK were assessed using Pearson’s product-moment correlation coefficient. Pearson’s correlation coefficients (r), corresponding 95% confidence intervals (CIs), and corresponding p-values were reported. To evaluate whether BMI was associated with radiation exposure factors such as procedural differences, separate multivariable regression models were not considered for DAP/KAP and CAK, incorporating BMI, procedure category, and patient variable recorded data in the study dataset. All 190 records represented individual patients, with each patient included once in the analysis.

The study was approved by the Institutional Ethics Committee (IEC), Srimanta Sankaradeva University of Health Sciences (SSUHS), Assam. The study proposal was approved at the IEC meeting held on April 27, 2026. The approval/order number was SSUHS/164/2016/Pt./2904; the forwarding order for the IEC minutes was dated June 4, 2026, and research-related data extraction or activities began after April 27, 2026, after getting the approval from the ethical committee.

## Results

A total of 190 archived patient records were obtained. The mean age of the cohort was 55.88 ± 11.04 years, and the median age was 57 years (IQR: 49-64; range: 10-80). The mean BMI was 26.78 ± 3.87 kg/m², and the median BMI was 26.55 kg/m² (IQR: 24.02-29.78; range: 18.9-39.3). Adult cutoffs were used for adult BMI. For pediatric patients, BMI-for-age Z-scores (5-19 years) were used, based on the 2007 WHO reference. A normal BMI was defined as between -2 SD and +1 SD, overweight as above +1 SD up to +2 SD, obesity as above +2 SD, thinness as below -2 SD down to -3 SD, and severe thinness as below -3 SD. The study population comprised 149 males (78.4%) and 41 females (21.6%) (Table 1).

**Table 1:** Demographic characteristics of the study population SD: standard deviation; IQR: interquartile range; BMI: body mass index

Variable	Value
Sample size	190
Age, years, mean ± SD	55.88 ± 11.04
Age, years, median (IQR)	57 (49–64)
Age range, years	10–80
BMI, kg/m², mean ± SD	26.78 ± 3.87
BMI, kg/m², median (IQR)	26.55 (24.02–29.78)
BMI range, kg/m²	18.9–39.3
Male, n (%)	149 (78.4%)
Female, n (%)	41 (21.6%)

CAG + PTCA was the most frequently performed procedure (70.5%), followed by diagnostic CAG (26.3%). Plain old balloon angioplasty and device closure together accounted for six cases (3.2%) (Table 2).

**Table 2:** Distribution of Cath Lab procedures Cath Lab: catheterization laboratory; CAG: coronary angiography; PTCA: percutaneous transluminal coronary angioplasty; POBA: plain old balloon angioplasty

Procedure	N	Percentage
CAG	50	26.3%
CAG + PTCA	134	70.5%
POBA	4	2.1%
Device closure	2	1.1%
Total	190	100%

Diagnostic CAG demonstrated the lowest median radiation exposure. CAG + PTCA showed substantially higher DAP/KAP and CAK, reflecting greater procedural complexity. The other procedures (POBA + device closure) group had the highest median DAP/KAP and CAK, but interpretation is limited by its small sample size (Table 3).

**Table 3:** Radiation exposure by procedure DAP: dose area product; CAK: cumulative air kerma; CAG: coronary angiography; PTCA: percutaneous transluminal coronary angioplasty; POBA: plain old balloon angioplasty

Procedure	N	DAP, Gy·cm², median (Q1–Q3)	DAP range, Gy·cm²	CAK, mGy, median (Q1–Q3)	CAK range, mGy
CAG	50	9.7 (7.0–14.6)	3.6–47.9	90.7 (63.3–138.2)	9.7–645.4
CAG + PTCA	134	32.0 (23.4–41.4)	6.1–158.0	382.1 (247.8–531.1)	20.8–1762.3
Other procedures (POBA + device closure)	6	36.1 (21.0–53.8)	6.8–73.3	612.6 (236.1–636.2)	63.5–1111.9
Overall	190	25.4 (14.1–37.7)	3.6–158.0	305.1 (135.4–475.9)	9.7–1762.3

Median fluoroscopy times were longest in other procedures (POBA+ device closure), followed by CAG + PTCA, and lowest in CAG (Table 4).

**Table 4:** Fluoroscopy time by procedure CAG: coronary angiography; PTCA: percutaneous transluminal coronary angioplasty; POBA: plain old balloon angioplasty

Procedure	N	Median (Q1–Q3), minutes	Range, minutes
CAG	50	1.9 (1.4–2.9)	0.9–12.4
CAG + PTCA	134	8.3 (5.7–11.6)	1.5–23.9
Other procedures (POBA + device closure)	6	13.1 (7.6–18.4)	1.8–21.2
Overall	190	6.2 (2.7–10.1)	0.9–23.9

Radiation exposure increased across BMI categories (Table 5). Median DAP rose from 16.8 Gy·cm² in the normal-BMI group to 25.4 Gy·cm² in the pre-obese group and 41.3 Gy·cm² in obesity class I. Median CAK similarly increased from 207.2 mGy to 300.2 mGy and 507.4 mGy, respectively. However, this trend was not strictly progressive, as the median values in obesity class II were slightly lower than those in obesity class I. This finding should be interpreted cautiously, given the small number of patients in obesity class II (n = 5).

**Table 5:** BMI-stratified radiation exposure BMI: body mass index; DAP: dose area product; CAK: cumulative air kerma

BMI category	N	DAP mean/median, Gy·cm²	DAP range	CAK mean/median, mGy	CAK range
Normal (18.5–24.9)	69	21.6/16.8	3.6–132.0	256.8/207.2	9.7–1287.6
Pre-obese (25.0–29.9)	78	27.8/25.4	4.4–86.0	328.3/300.2	20.8–1111.9
Obesity class I (30.0–34.9)	38	46.4/41.3	6.9–158.0	568.7/507.4	66.5–1762.3
Obesity class II (35.0–39.9)	5	47.2/39.5	34.7–69.8	527.4/492.0	231.9–731.8

Our Pearson’s correlation analysis showed an unadjusted, moderate positive correlation between BMI and DAP/KAP (r = 0.470, 95% CI: 0.351-0.574, p < 0.001) and between BMI and CAK (r = 0.469, 95% CI: 0.350-0.573, p < 0.001). It also showed a strong positive correlation between the two radiation indicators, DAP/KAP and CAK (r = 0.941, 95% CI: 0.922-0.955, p < 0.001) (Table 6).

**Table 6:** Pearson’s correlation between BMI and radiation exposure parameters BMI: body mass index; CI: confidence interval; DAP: dose area product; KAP: kerma air product; CAK: cumulative air kerma

Variables compared	Pearson’s correlation coefficient (r)	95% CI	P-value	Interpretation
BMI and DAP/KAP	0.47	0.351–0.574	< 0.001	Moderate positive correlation
BMI and CAK	0.469	0.350–0.573	< 0.001	Moderate positive correlation
DAP/KAP and CAK	0.941	0.922–0.955	< 0.001	Very strong positive correlation

Procedure-wise Pearson’s correlation analysis showed positive associations between BMI and radiation exposure across all three procedural categories. In the CAG group, BMI showed a significant positive correlation with DAP/KAP (r = 0.312, 95% CI: 0.040-0.541, p = 0.026) and CAK (r = 0.324, 95% CI: 0.050-0.553, p = 0.020). Similarly, in the CAG + PTCA group, significant positive correlations were observed between BMI and DAP/KAP (r = 0.363, 95% CI: 0.205-0.502, p < 0.001) and CAK (r = 0.354, 95% CI: 0.194-0.495, p < 0.001). In the other-procedure group, comprising POBA and atrial septal defect (ASD) device closure, strong positive correlations were observed for DAP/KAP (r = 0.879, 95% CI: 0.235-0.987, p = 0.021) and CAK (r = 0.914, 95% CI: 0.397-0.991, p = 0.011) (Table 7). However, these findings should be interpreted cautiously because this subgroup included only six patients. In addition, the unequal distribution of patients across BMI and procedural subgroups, particularly obesity class II (n = 5), should be considered when interpreting the results. The small sample sizes may produce unstable estimates and limit the generalizability of subgroup findings.

**Table 7:** Procedure-wise pearson's correlation between BMI and radiation exposure BMI: body mass index; DAP: dose-area product; KAP: kerma-area product; r: Pearson’s correlation coefficient; CI: confidence interval; CAK: cumulative air kerma; CAG: coronary angiography; PTCA: percutaneous transluminal coronary angioplasty; POBA: plain old balloon angioplasty; ASD: atrial septal defect

Procedure category	N (DAP)	BMI vs. DAP/KAP (r)	95% CI	P-value	N (CAK)	BMI vs. CAK (r)	95% CI	P-value
CAG	50	0.312	0.040 to 0.541	0.026	50	0.324	0.050–0.553	0.02
CAG + PTCA	134	0.363	0.205 to 0.502	< 0.001	134	0.354	0.194–0.495	< 0.001
Other (POBA + ASD device closure)	6	0.879	0.235 to 0.987	0.021	6	0.914	0.397–0.991	0.011

No radiation-dose alerts or immediate radiation-related complications were documented in the archived procedural records and also the systematic post-procedural follow-up specifically assessing delayed radiation-induced skin injuries was not performed in the archived dataset. Furthermore, institutional radiation-dose thresholds for triggering alerts were also not available in the records.

## Discussion

The present study is a retrospective, single-center study that evaluated archived cardiac cath lab data and radiation exposure parameters among 190 patients undergoing CAG, CAG + PTCA, and other procedures, namely device closure and POBA, over six months. CAG + PTCA was the most commonly performed procedure (70.5%), followed by diagnostic CAG alone (26.3%) and other procedures (3.2%) (Table 2).

Radiation exposure parameters (DAP/KAP, CAK/Ka,r, and fluoroscopy time) were archived and compared among the procedures carried out in the cardiac cath lab. Diagnostic CAG had the lowest radiation exposure, whereas CAG + PTCA had substantially higher radiation exposure (Table 3). This pattern is clinically plausible because interventional procedures in cardiology generally require longer fluoroscopy, repeated image acquisition, and additional device manipulation than diagnostic angiography and are therefore associated with greater exposure to ionizing radiation. Anim-Sampong et al. reported similar findings and emphasized radiation optimization [13]. The observed variation also supports the value of procedure-specific radiation recording rather than reliance on a single overall institutional dose estimate [5,7,8].

The other-procedures group (POBA and device closure) had the highest median DAP/KAP and CAK and the longest median fluoroscopy time (Tables 3, 4). However, this finding should be interpreted cautiously because the group contained only six cases and combined clinically heterogeneous procedures. A small number of complex procedures can markedly influence the median dose, so this result should be regarded as an institutional observation rather than evidence that these procedures consistently carry the highest radiation burden. Nevertheless, the finding illustrates the importance of reviewing radiation exposure by procedure type and complexity [5,7-13].

The relationship between fluoroscopy time and radiation dose is clinically important. The present study showed longer fluoroscopy times in the more complex procedure categories compared with diagnostic CAG (Table 4). Fluoroscopy duration, cine acquisitions, imaging settings, and operator technique are recognized contributors to radiation exposure during fluoroscopy-guided cardiac procedures [5,7,10]. Thus, fluoroscopy time can serve as a practical screening indicator for procedures that may warrant closer radiation review, although it should not be interpreted as a complete surrogate for patient dose because DAP/KAP and CAK are also influenced by field size, patient habitus, and exposure settings [14].

The observed increase in radiation exposure across the lower and intermediate BMI categories suggests an association between patient body habitus and radiation requirements. However, these findings should be interpreted in the context of potential procedural and technical confounding factors (Table 5). This finding is consistent with the established effect of patient body habitus on fluoroscopic radiation requirements: increased tissue thickness may require higher X-ray output to maintain adequate image quality [10-12,15]. The stepwise increase in median DAP/KAP and CAK across the BMI categories in the present dataset therefore has practical relevance for radiation-management strategies. Patients were not followed up. Occupational dosimetry was not measured in this study [15].

The moderate positive correlations observed between BMI and both DAP/KAP (r = 0.470) and CAK (r = 0.469) (Table 6) further support the importance of considering patient-specific body habitus during radiation management. These findings indicate an association rather than causation. BMI may influence radiation output, but radiation exposure is also affected by procedure type and complexity, fluoroscopy duration, cine acquisition, imaging settings, and operator-related factors [5-7,10-12,15]. Consequently, BMI should be considered as one component of a broader patient- and procedure-specific radiation optimization strategy, an approach that has been adopted by many centers.

The very strong positive correlation between DAP/KAP and CAK (r = 0.941) indicates a close association between these complementary radiation indicators. Although closely associated, they represent distinct dosimetric parameters with different units and clinical interpretations. DAP/KAP reflects radiation burden integrated over the irradiated area, whereas CAK/Ka,r reflects radiation at the interventional reference point and is particularly relevant when considering potential deterministic skin effects [7-9]. Recording both measures therefore provides complementary information and may improve the ability of the cardiac cath lab archive to identify procedures requiring radiation review.

Procedure-wise analysis showed significant positive correlations between BMI and both DAP/KAP and CAK across all procedural categories. These associations were relatively consistent in the CAG and CAG + PTCA groups, while stronger correlations were observed in the other-procedure group (POBA and ASD device closure). However, the latter findings should be interpreted cautiously because of the small sample size (n = 6). Additionally, the unequal distribution of patients across BMI categories, particularly obesity class II (n = 5), may have affected the stability and precision of subgroup estimates. Therefore, these findings should be interpreted cautiously, and larger studies are needed to validate the observed associations (Table 7).

An important practical contribution of the study is the establishment of an institutional baseline. Systematic recording of radiation-dose parameters can help identify unusually high-dose procedures, compare radiation burden between procedure types, support protocol optimization, and facilitate quality-improvement initiatives [6,9]. A structured archive also permits longitudinal review of dose patterns and can support clinical auditing, performance benchmarking, and future research. The present findings therefore show how routine digital archiving can be used not only for record keeping but also as a practical radiation-safety and quality-assurance tool.

Study limitations

This study has several limitations. Its retrospective, single-center design relied on routinely archived records, making the findings dependent on the completeness and consistency of recorded clinical and radiation data. The six-month study period and sample size of 190 may limit statistical precision and external generalizability. The small, heterogeneous “other procedures” subgroup (n = 6) and the small number of patients in obesity class II (n = 5) also limit reliable interpretation of the radiation-dose estimates. Although BMI was significantly associated with DAP/KAP and CAK, the analysis did not fully adjust for procedural complexity, lesion characteristics, access route, operator experience, imaging projections, or frame-rate settings, leaving the possibility of residual confounding. BMI alone may also incompletely represent patient body habitus and radiation attenuation. Finally, the observational nature of the study means that the observed associations should not be interpreted as causal.

The study did not identify radiation-related clinical complications during the study period. Nevertheless, the absence of documented deterministic effects should not be interpreted as absence of radiation risk. Continued monitoring of dose indicators and adherence to the ALARA principle remain important components of radiation protection [5,6,9,12]. In this context, maintaining complete records of DAP/KAP, CAK/Ka,r, and fluoroscopy time can facilitate early identification of unusually high exposures and support appropriate review of procedural practice.

## Conclusions

Radiation exposure during cardiac catheterization procedures varied considerably by procedure type and patient BMI. Diagnostic CAG was associated with the lowest radiation exposure, whereas combined CAG + PTCA was associated with higher radiation exposure. BMI showed a significant positive association with both DAP/KAP and CAK, while DAP/KAP and CAK showed a very strong positive correlation. These findings emphasize the importance of recording multiple indicators of radiation exposure rather than relying on fluoroscopy time alone. Our study established a structured archive of Cath Lab procedural and radiation exposure data. Such systematic documentation can support radiation safety monitoring, procedural evaluation, quality assurance, dose optimization, clinical auditing, and future research. The institutional baseline generated by this study may serve as a foundation for continuous radiation surveillance and improvement of Cath Lab practice.
